# Acute Myocardial Infarction Following Tenecteplase: A Case Report of Complex Dual Pathology

**DOI:** 10.5811/cpcem.64754

**Published:** 2026-10-20

**Authors:** Devin Howell, Ramses Ramirez Damera, Gaurav Rao

**Affiliations:** * Northwell Health, New Hyde Park, New York; † Donald and Barbara Zucker School of Medicine, Hempstead, New York; ‡ Huntington Hospital, Department of Emergency Medicine, Huntington, New York; § Nassau University Medical Center, East Meadow, New York; ¶ Huntington Hospital, Department of Cardiology, Huntington, New York

**Keywords:** stroke, STEMI, thrombolytics, tenecteplase, case report

## Abstract

**Introduction:**

Acute ischemic stroke is a leading cause of morbidity and mortality, with tenecteplase being a cornerstone of acute management. While tenecteplase carries a known risk of bleeding, major thrombotic events such as acute myocardial infarction (AMI) after stroke reperfusion are less commonly described.

**Case Report:**

We present the case of a 67-year-old man who presented with acute stroke symptoms. After ruling out intracranial hemorrhage and managing blood pressure, he received tenecteplase, and all neurological deficits resolved. Soon after, he developed acute chest pain, and his repeat electrocardiogram confirmed a ST-elevation myocardial infarction. He underwent emergent cardiac catheterization, revealing 100% occlusion of the left circumflex artery, which was successfully revascularized with thrombectomy and angioplasty. Intravascular ultrasound further supported the presence of embolic phenomenon. Pathology of the retrieved material identified thrombus alongside atherosclerotic plaque fragments. The patient experienced complete symptom resolution and was discharged after a short hospitalization.

This case highlights the complexities encountered when a patient with acute ischemic stroke who is treated with tenecteplase subsequently develops an AMI. Potential mechanisms include a coincidental plaque rupture, possibly exacerbated by the stress of the acute stroke, a rare paradoxical prothrombotic effect of thrombolysis, or a combination thereof. The pathological finding of mixed thrombus and atherosclerotic plaque underscores the underlying disease contributing to this event.

**Conclusion:**

This case accentuates the importance of maintaining a high index of suspicion for new cardiovascular events in post-thrombolysis patients, especially those with significant cardiovascular risk factors. Rapid and judicious diagnostic evaluation is crucial for timely management of these complex, life-threatening complications.

## INTRODUCTION

Acute ischemic stroke remains a leading cause of long-term disability and death worldwide.^1^ Rapid reperfusion therapies, including intravenous thrombolysis with agents such as tenecteplase are critical for improving neurological outcomes by restoring blood flow to ischemic brain tissue.^2^ Tenecteplase, a modified recombinant tissue plasminogen activator, offers practical advantages over alteplase, including a single bolus administration, and has demonstrated noninferiority and potential superiority in certain contexts.^3^,^4^ While tenecteplase is highly effective, its use carries an inherent risk of bleeding complications, most notably intracranial hemorrhage.^2^ Less frequently reported are major arterial thrombotic events distant from the initial cerebrovascular occlusion, such as acute myocardial infarction, which can present a significant diagnostic and therapeutic challenge in the post-thrombolysis setting.^5^

This case report describes a unique scenario where a patient achieved complete neurological recovery following tenecteplase administration for acute ischemic stroke, only to subsequently develop an acute posterolateral ST-elevation myocardial infarction (STEMI). The pathology of the retrieved coronary thrombus revealed a combination of thrombus and calcified atherosclerotic plaque, providing critical insights into the potential underlying mechanisms. This report highlights the diagnostic complexities, discusses potential pathophysiological considerations, and emphasizes the clinical vigilance required in managing such intricate cases.

## CASE REPORT

A 67-year-old man with a medical history significant for hypertension, hyperlipidemia, a known cerebral aneurysm status postcoiling, a thoracic aortic aneurysm (stable, nonsurgical), and gastroesophageal reflux disease, presented to the emergency department with acute onset left-sided weakness, slurred speech, and facial droop. His symptom onset was approximately one hour prior to arrival.

Upon presentation, his vital signs were as follows: heart rate, 84 beats per minute; blood pressure, 187/121 mm Hg; respiratory rate, 18 breaths per minute; SpO_2_, 98% on room air; and blood glucose 89 mg/dL (reference range, 70**–**99 mg/dL). Neurological examination revealed a National Institutes of Health Stroke Scale (NIHSS) score of 10, characterized by partial paralysis of the left face (two points), left arm and leg hemiparesis with no effort against gravity (arm = three points, leg = three points), and severe dysarthria (two points). He was activated as a code stroke based on hospital policy.

Noncontrast computed tomography of the brain no evidence of acute intracranial hemorrhage or mass effect. A subsequent computed tomography angiogram (CTA) of the head and neck did not reveal any large vessel occlusion amenable to endovascular thrombectomy, and his known cerebral aneurysm appeared stable. Laboratory workup, including a complete blood count, complete metabolic panel, and coagulation studies, were within reference range, with an initial high-sensitivity troponin being undetectable. Following blood pressure control using a single push-dose of intravenous (IV) labetalol to achieve parameters below 185/110 mm Hg, the patient received a single bolus of tenecteplase (0.25 mg/kg) approximately 95 minutes from symptom onset. An IV infusion of nicardipine IV was initiated to maintain blood pressure below 180/105 mm Hg.

*CPC-EM Capsule*

What do we already know about this clinical entity?

*Tenecteplase is commonly administered for stroke, ST-elevation myocardial infarction (STEMI), and pulmonary embolism (PE).*

What makes this presentation of disease reportable?

*Thromboembolic events following tenecteplase administration are rare but life-threatening.*

What is the major learning point?

*Patients receiving tenecteplase should be monitored for hemorrhage and thromboembolic events, including STEMI, PE, or peripheral arterial obstruction.*

How might this improve emergency medicine practice?

*Thrombolytic administration is common when time to catheterization is >120 minutes. Readers should be aware of this rare complication of tenecteplase.*

Within 15 minutes of tenecteplase administration, the patient exhibited significant neurological improvement. A repeat neurological assessment demonstrated complete resolution of his left-sided weakness, facial droop, and dysarthria, with a subsequent NIHSS score of 0. Approximately 30 minutes after tenecteplase administration, the patient developed sudden, severe, retrosternal chest pain radiating to his left arm, accompanied by diaphoresis. An immediate 12-lead electrocardiogram (ECG) revealed massive ST segment depressions in leads V1**–**V6 (Image 1).

A posterior 12-lead ECG confirmed ST-segment elevations in V7**–**V9, indicating an acute posterolateral STEMI. Serial troponin levels rapidly rose, peaking at over 8,000 ng/L (reference range, < 20 ng/L). Given his history of an aortic aneurysm, an immediate CTA of the chest was performed to rule out aortic dissection, which resulted as negative. The patient was not given antiplatelets or heparin in the setting of recent tenecteplase administration. He was emergently transferred to the cardiac catheterization laboratory for further management.

Coronary angiography revealed a 100% thrombotic occlusion of the mid-left circumflex artery (Image 2).

The culprit lesion was successfully wired, and a small-balloon angioplasty was performed to create a channel. This was followed by aspiration thrombectomy using a catheter to remove significant thrombotic material (Image 3), resulting in thrombolysis in myocardial infarction grade 3 flow and ST-segment resolution.

Intravascular ultrasound was used to characterize the culprit site, revealing a lack of significant underlying atherosclerotic burden or evidence of a ruptured fibroatheroma. Due to the favorable angiographic result and absence of a high-grade stenotic lesion, stent placement was deferred. Pathological analysis of the retrieved revealed the presence of a thrombus extensively intermingled with calcified atherosclerotic plaque fragments. This finding strongly suggested mobilization and acute plaque rupture of aortic or proximal arterial debris as the underlying mechanism for the STEMI.

Following successful revascularization, the patient’s chest pain resolved. His postprocedure course in the intensive care unit was uneventful. A transthoracic echocardiogram revealed normal left ventricular function without regional wall motion abnormalities corresponding to the lateral and posterior walls, and an ejection fraction of 56%. He was initiated on guideline-directed medical therapy, including aspirin, high-intensity statin, beta blocker, and an angiotensin-II receptor blocker. The patient remained neurologically intact, with no recurrence of stroke symptoms or bleeding complications. He was discharged on hospital day 6 in stable condition with close outpatient follow-up arranged for both his cardiac and neurological conditions.

## DISCUSSION

This case highlights a rare but critical sequence of events: an elderly man with multiple cardiovascular risk factors who experienced an acute ischemic stroke with complete resolution following tenecteplase, only to develop an acute posterolateral STEMI shortly thereafter. This complex presentation underscores the challenges in diagnosing and managing concurrent or sequential cardiovascular and cerebrovascular events, particularly in the context of recent thrombolytic therapy.

The initial neurological presentation and rapid response to tenecteplase were textbook examples of successful thrombolysis for acute ischemic stroke. However, the subsequent development of STEMI presented a significant diagnostic dilemma. Given the patient’s history of an aortic aneurysm, aortic dissection was a primary concern, necessitating a prompt aortic CTA to rule out this life-threatening condition. The immediate availability of a comprehensive diagnostic pathway, including serial electrocardiograms (ECG), troponin levels, and imaging, was crucial in differentiating the cause of chest pain and activating the appropriate “Code STEMI” pathway.

Several mechanisms could explain the occurrence of STEMI in this patient. His extensive cardiovascular risk factors predisposed him to advanced atherosclerosis. The pathological finding of thrombus with associated calcified atherosclerotic plaque with normal coronary artery architecture strongly supports the hypothesis of embolic phenomenon as the primary event leading to left circumflex artery occlusion. The acute stress associated with the stroke, including sympathetic surge, inflammatory response, and hemodynamic fluctuations, could have further destabilized a vulnerable plaque, leading to its rupture.^6^

While thrombolytics like tenecteplase are designed to dissolve clots, there have been theoretical discussions and rare case reports suggesting a potential paradoxical prothrombotic effect or rebound hypercoagulable state following systemic thrombolysis.^5^,^7^,^8^ This could occur through various mechanisms, such as increased thrombin generation, platelet activation, or depletion of natural anticoagulants. In a patient with preexisting unstable atherosclerotic plaque, such a state could potentially precipitate or exacerbate acute thrombus formation at another site.

While intracranial hemorrhage is the most feared bleeding complication of thrombolysis, new arterial thrombotic events distant from the original clot, such as myocardial infarction, are considerably rarer. A search of the medical literature revealed a few case reports rather than large-scale studies.^5^,^9^ One review highlighted cases of acute limb ischemia or myocardial infarction occurring after systemic thrombolysis for pulmonary embolism or stroke, attributing some to paradoxical hypercoagulability but acknowledging the challenge in differentiating from coincidental events.^5^ We identified only two other case reports that described a STEMI occurring after tenecteplase for stroke, where the underlying mechanism was also attributed to plaque rupture.^8^,^9^ Our case adds to this limited body of evidence, providing the unique pathological confirmation of underlying atherosclerotic plaque alongside thrombus in the retrieved material without histological evidence of coronary disease on imaging. This suggests that while systemic hemostatic changes induced by tenecteplase cannot be entirely excluded, the primary event was likely related to the patient’s preexisting and highly vulnerable aortic atherosclerosis leading to coronary embolism.

This case highlights several critical clinical implications. First, a high index of suspicion for new cardiovascular events must be maintained in post-thrombolysis stroke patients, even in the setting of successful neurological recovery. Patients with multiple cardiovascular risk factors are particularly vulnerable. Second, rapid and systematic evaluation, including serial ECGs, troponin measurements, and judicious use of imaging (ie, CTA to rule out aortic dissection), is paramount to distinguish new symptoms from other potential complications or coincidental events. Finally, the management of acute coronary syndromes in the context of recent thrombolysis requires careful consideration of bleeding risk versus ischemic risk, particularly when deciding on antithrombotic and antiplatelet regimens and the approach to revascularization. In our case, the decision to defer stent placement in favor of thrombectomy and balloon angioplasty reflected an attempt to minimize further potent antiplatelet requirements and bleeding risk in the immediate post-tenecteplase period.

## LIMITATIONS

This is a single case report, limiting the generalizability of our findings. While the pathological findings provide strong evidence for coronary embolization, it is impossible to definitively determine the exact interplay between tenecteplase administration, the acute stroke, and the timing of the myocardial infarction without a larger cohort of similar cases.

## CONCLUSION

This case report describes a unique and challenging clinical scenario of an acute STEMI succeeding successful tenecteplase administration for an acute ischemic stroke. The pathological and IVUS findings of thrombus with associated calcified plaque fragments in an otherwise non-obstructive vessel support an embolic mechanism, likely from an aortic source. This case underscores the need for vigilance regarding multi-territory embolic events in patients with a high burden of systemic atherosclerosis undergoing thrombolysis. Rapid diagnostic evaluation and multidisciplinary management are essential for optimizing outcomes in these complex patients.

## Figures and Tables

**Image 1:**
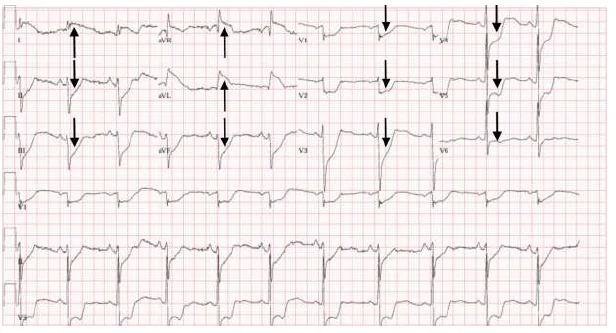
Electrocardiogram performed with initial onset of chest pain. Depressions noted in leads II, III, aVF, and V1–V6 with elevation in leads I, aVR, and aVL

**Image 2:**
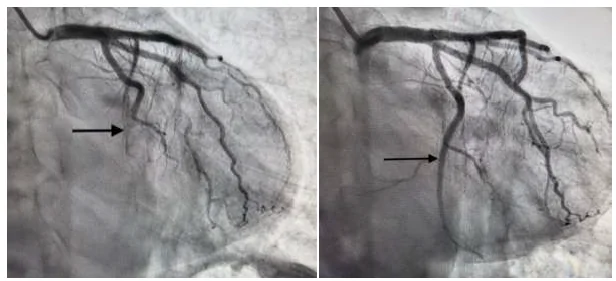
Coronary angiography of the left main coronary artery showing 100% occlusion of the left circumflex artery (left). Coronary angiography of the left main coronary artery after thrombectomy with thrombolysis in myocardial infarction 3 flow (right).

**Image 3:**
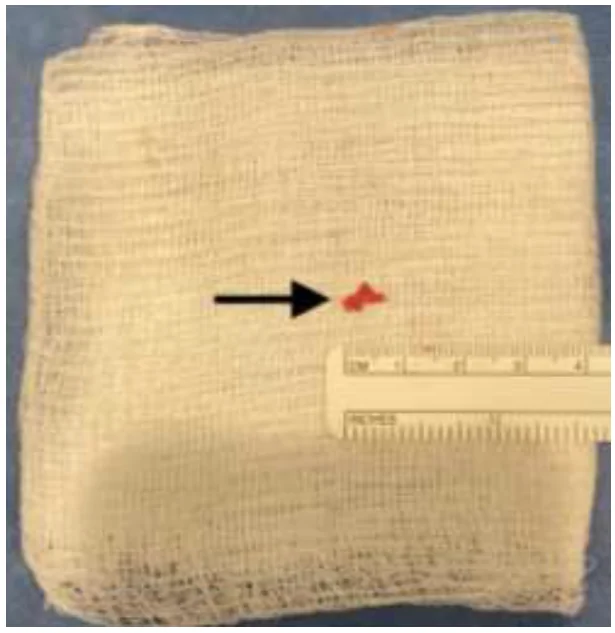
Thrombotic atherosclerotic plaque removed during coronary angiography using a catheter.
